# Adrenomedullin in COVID-19 induced endotheliitis

**DOI:** 10.1186/s13054-020-03151-7

**Published:** 2020-07-09

**Authors:** Darius Cameron Wilson, Joerg C. Schefold, Jaume Baldirà, Thibaud Spinetti, Kordo Saeed, Gunnar Elke

**Affiliations:** 1 Shock, Organ Dysfunction and Resuscitation Research Group, Vall d’Hebron Institut of Research, Barcelona, Spain; 2 Department of Intensive Care Medicine, Inselspital, Bern University Hospital, University of Bern, Bern, Switzerland; 3 Intensive Care Department, Hospital de la Santa Creu i Sant Pau, Barcelona, Spain; 4 University Hospital Southampton NHS Foundation Trust, Southampton, UK; 5 Department of Anaesthesiology and Intensive Care Medicine, University Medical Center Schleswig-Holstein, Campus Kiel, Arnold-Heller-Str. 3 Haus R3, 24105 Kiel, Germany

Despite the exponential growth in research following the rapid spread of SARS-CoV-2 and subsequent COVID-19 pandemic, the underlying pathophysiological mechanisms in COVID-19 patients remain poorly understood. The increased incidence of cardiovascular and thromboembolic complications, immune cell deactivation and sepsis-like multiple organ failure suggests the involvement of multiple pathways. Accordingly, recent studies have proposed that virus-induced endothelial dysfunction and damage, resulting in impaired vascular blood flow, coagulation and leakage, may partially explain the development of organ dysfunction and oedema [1]. Hence, the development of endotheliitis may be a prominent, yet partly under recognised, feature of COVID-19 induced critical illness.

Whilst numerous pro-inflammatory cytokines and blood biomarkers have already been compared in patients with different severities of COVID-19 - to date - no study, report or editorial has described the potential role of adrenomedullin (ADM) during the host response to COVID-19. This is surprising, since ADM has been shown to play a key role in reducing vascular (hyper) permeability and promoting endothelial stability and integrity following severe infection [2]. Thus, ADM may also be of interest within COVID-19 induced endotheliitis. Indeed, a recent study investigating gene upregulation in patients with systemic capillary leak syndrome (SCLS), characterised by plasma leakage into peripheral tissue and transient episodes of hypotensive shock and oedema, found that ADM was not only one of the most upregulated genes, but that subsequent application to endothelial cells resulted in a protective effect on vascular barrier function [3].

Furthermore, recent clinical studies on sepsis patients upon emergency department (ED) presentation and during intensive care (ICU) treatment using the stable protein surrogate, mid-regional proadrenomedullin (MR-proADM), found that its assessment could accurately identify disease progression in patients with non-severe clinical signs and symptoms, safely increase out-patient treatment with decreased readmission rates and no subsequent mortalities [4], and identify patients requiring a rapid administration of antibiotics or triage to the ICU [5]. Despite the low number of severe viral cases within each of these studies (between 2.1% [3] and 3.4% [4]), similar hypotheses can also be formulated for patient populations with COVID-19.

The assessment of MR-proADM in future COVID-19 studies may therefore provide important information into the pathophysiological mechanisms underlying endotheliitis and subsequent organ dysfunction. The early identification of patients likely to develop severe clinical symptoms requiring subsequent hospitalisation, as well as the safe discharge of those already hospitalised, may be of particular importance in regions where healthcare systems are used to full capacity.

## Data Availability

Not applicable

## Abbreviations

ADM: Adrenomedullin
ED: Emergency department
ICU: Intensive care unit
MR-proADM: Mid-regional proadrenomedullin
SARS-CoV-2: Severe acute respiratory syndrome coronavirus 2
SCLS: Systemic capillary leak syndrome
